# Folic Acid Confers Tolerance against Salt Stress-Induced Oxidative Damages in Snap Beans through Regulation Growth, Metabolites, Antioxidant Machinery and Gene Expression

**DOI:** 10.3390/plants11111459

**Published:** 2022-05-30

**Authors:** Hameed Alsamadany, Hassan Mansour, Amr Elkelish, Mohamed F. M. Ibrahim

**Affiliations:** 1 Department of Biological Sciences, Faculty of Science, King Abdulaziz University, Jeddah 21589, Saudi Arabia; 2 Department of Biological Sciences, College of Science and Arts, King Abdulaziz University, Rabigh 21911, Saudi Arabia; hmansor@kau.edu.sa; 3 Botany Department, Faculty of Science, Suez Canal University, Ismailia 41522, Egypt; amr.elkelish@science.suez.edu.eg; 4 Department of Agricultural Botany, Faculty of Agriculture, Ain Shams University, Cairo 11566, Egypt

**Keywords:** *Phaseolus vulgaris* L., folate biofortification, salt stress and membranes transporters

## Abstract

Although the effect of folic acid (FA) and its derivatives (folates) have been extensively studied in humans and animals, their effects are still unclear in most plant species, specifically under various abiotic stress conditions. Here, the impact of FA as a foliar application at 0, 0.1, and 0.2 mM was studied on snap bean seedlings grown under non-saline and salinity stress (50 mM NaCl) conditions. The results indicated that under salinity stress, FA-treated plants revealed a significant (*p* ≤ 0.05) increase in growth parameters (fresh and dry weight of shoot and root). A similar trend was observed in chlorophyll (Chl b), total chlorophyll, carotenoids, leaf relative water content (RWC), proline, free amino acids (FAA), soluble sugars, cell membrane stability index (CMSI), and K, Ca, and K/Na ratio compared to the untreated plants. In contrast, a significant decrease was observed in Na and salinity-induced oxidative damage as indicated by reduced H_2_O_2_ production (using biochemical and histochemical detection methods) and rate of lipid peroxidation (malondialdehyde; MDA). This enhancement was correlated by increasing the activities of antioxidant enzymes, i.e., superoxide dismutase (SOD), catalase (CAT), guaiacol peroxidase (G-POX), and ascorbate peroxidase (APX). Gene expression analyses conducted using qRT-PCR demonstrated that genes coding for the Na^+^/H^+^ antiporter protein Salt Overly Sensitive 1 (*SOS1*), the tonoplast-localized Na^+^/H^+^ antiporter protein (*NHX1*), and the multifunctional osmotic protective protein (*Osmotin*) were significantly up-regulated in the FA-treated plants under both saline and non-saline treatments. Generally, treatment with 0.2 mM FA was more potent than 0.1 mM and can be recommended to improve snap bean tolerance to salinity stress.

## 1. Introduction

Salinity is one of the most widespread abiotic stresses which results in significant losses in agricultural crop production, especially in arid and semi-arid areas [1]. Most of the cultivated plants are salt sensitive, and hence, they are prone to death and wilting because of salt toxicity [2]. It has been found that moderate salinity causes about 50–80% loss in the yield of 30 crop plant species which provide about 90% of plant-based human food [3]. Recently, the amount of salt-affected land was estimated as 1125 million hectares worldwide [4]. This area is expected to increase with frequent climate changes, increasing human activities, and freshwater limitations [5,6]. Salinity can induce different molecular, physiological, and biochemical malformations [7,8,9]. It inhibits plant growth and development by affecting plant water relations, cell turgor pressure, and disturbing plant growth regulators, restricting cell division and enlargement [10]. Additionally, it can negatively affect photosynthesis, transpiration, and stomatal conductance [11,12]. Furthermore, salinity stress disintegrates the cell membrane and disturbs ion homeostasis, leading to variations in cell ultrastructure and imbalances in nutrient uptake [9,13]. Several papers have indicated that salinity stress induced oxidative damage at the cellular level due to the excessive release of reactive oxygen species (ROS) in different plant species [8,9,11,14].

Vitamins produced by plants are essential, not only for their effect on humans when ingested but also for their vital role in the plant itself [15]. Some of them show strong antioxidant potential, such as the water-soluble vitamins (B and C) as well as the lipid-soluble ones (A, E, and K) [16]. In this context, vitamin B9 (folic acid; FA) and its derivatives (folates) are major players in the metabolism of both carbohydrates and nitrogen [17]. Being the main cofactors, folates can be involved in the methylation cycle and DNA biosynthesis [18], since reducing folates has been found to decrease the nucleotide production in *Arabidopsis thaliana* [19]. Hence, the lesser production of folates, the lesser cell division rate, genome stability, and growth [18]. Moreover, it has been confirmed that under different stress conditions, endogenous folates can be strongly affected in many plant species [18,20]. This effect may be due to the down-regulation of folate biosynthesis-related genes [21,22,23]. This response may highlight the importance of exogenous folates to maintain a better plant cell and organ state under diverse environmental stresses. Generally, folates can be involved in the biosynthesis of plant growth regulators i.e., auxins, polyamines, and ethylene, through their direct role in the biosynthesis of amino acids, such as methionine and glycine tryptophan, glutamic, and valine [17]. Furthermore, folates are indirectly responsible for the biosynthesis of chlorophyll, carotenoids, and α-tocopherol (vitamin E). These effects are due to their participation in the biosynthesis of porphyrins, S-adenosylmethionine (SAM), and isoprenoids [17,24]. Additionally, the strong antioxidant properties of folates can be considered a key factor in elucidating their role in enhancing plant tolerance to diverse abiotic stresses and preventing oxidative damage [25,26]. In this context, exogenous FA has an important role in alleviation the harmful effects of abiotic stresses such as drought [24,27] and salinity [28].

Snap bean (*Phaseolus vulgaris* L.) represents 50% of legume consumption as a human food source worldwide [29]. It contains fibers, vitamins, and micronutrients, and has a high protein content [24,30]. Many epidemiological studies have shown positive correlations between legume (especially bean)-rich diets and increased longevity [31], improved heart condition [32,33], and reduced risks for many types of cancers [34,35]. On the other hand, snap bean is considered a salt-sensitive crop (glycophyte), with a threshold salinity level of 1 dS m^−1^ [36]. Hence, having more salt-tolerant plants or alleviating the harmful effects of salinity stress is extremely important to get a sufficient yield at the economic level.

Up to now, the effect of exogenous FA on snap bean plants grown under saline conditions has received very little attention. Therefore, this study was conducted to better understand the possible roles of FA as a protective mediator to attenuate the salt-induced oxidative damage in snap bean plants and enhance their tolerance to salinity stress.

## 2. Results

### 2.1. Effect of FA on Vegetative Growth under Saline and Non-Saline Conditions

**Figure 1:**
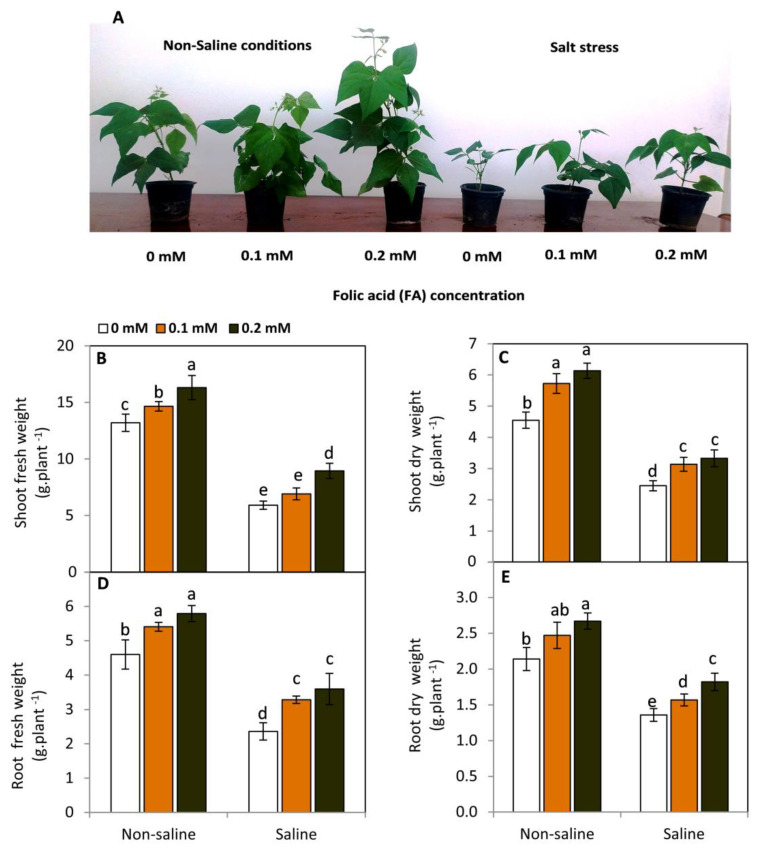
Effect of exogenous folic acid (FA; 0, 0.1, and 0.2 mM) on vegetative growth (**A**), shoot fresh weight (**B**), shoot dry weight (**C**), root fresh weigh (**D**) and root dry weight (**E**), of snap bean plants grown under non-stressed and saline-stressed (50 mM, NaCl) conditions. Values are the averages of three replicates ± SD. Different letters indicate significant differences according to Duncan’s multiple range tests (*p* < 0.05).

Data presented in Figure 1 show that plants exposed to salt stress (50 mM NaCl) demonstrated a significant decline (*p* ≤ 0.05) in their growth rate compared to the unstressed plants. In contrast, the foliar application of FA led to an obvious and significant (*p* ≤ 0.05) improvement in growth either under saline or non-saline conditions. This positive effect was explicit as a direct result of enhancing the fresh and dry weights of the shoot and root systems. Under saline conditions, the highest significant increase in shoot fresh and dry weights was achieved by the treatment of FA at 0.2 mM. This increase was 51.2 and 35.9%, respectively, compared to the untreated plants. A similar trend was observed in the root fresh (52.1%) and dry weight (33.8%).

### 2.2. Effect of FA on Leaf Photosynthetic Pigments

**Figure 2:**
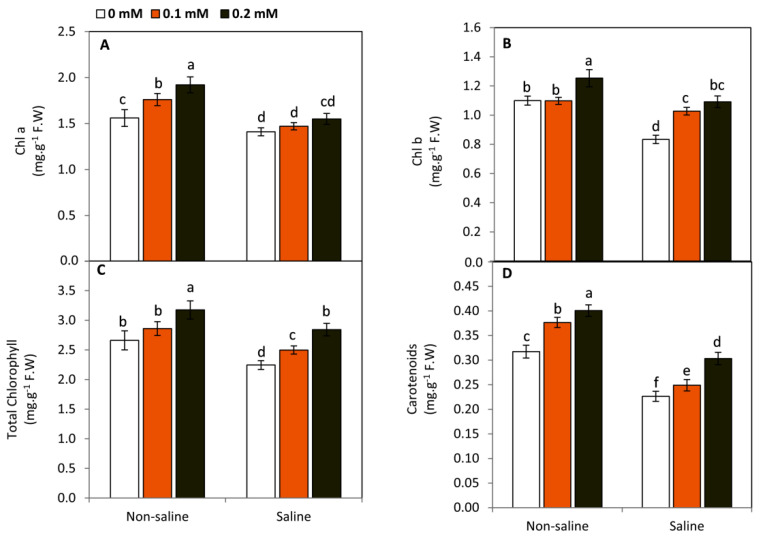
Effect of exogenous folic acid (FA; 0, 0.1, and 0.2 mM) on photosynthetic pigments including Chl a (**A**), Chl b (**B**), total chlorophyll (**C**), and carotenoids (**D**) of snap bean plants grown under non-stressed and saline-stressed (50 mM, NaCl) conditions. Values are the averages of three replicates ± SD. Different letters indicate significant differences according to Duncan’s multiple range tests (*p* < 0.05).

Plants exposed to saline conditions exhibited a significant (*p* ≤ 0.05) decrease in the concentration of photosynthetic pigments, including Chl a, Chl b, Chl a + b, and carotenoids, compared to the non-saline conditions (Figure 2). Under saline conditions, FA-treated plants demonstrated an obvious and significant improvement in Chl b, Chl a + b, and carotenoids compared to the untreated plants under saline conditions, the highest significant values (30.9, 26.6, and 34%) were achieved by 0.2 mM FA-treatment in Chl b, Chl a + b, and carotenoids, respectively. Meanwhile, no significant changes were detected in Chl a of FA-treated and non-treated plants under saline conditions. On the other hand, FA-treated plants, specifically at 0.2 mM, demonstrated an obvious and significant increase in all studied leaf photosynthetic pigments compared to the non-treated plants. These results imply that FA treatments may protect the photosynthetic apparatus under saline conditions by reinforcing Chl b and carotenoids. These responses can be considered an important regulatory step to avoid the exceeding light absorbance and restrict the over reduction of photosynthetic electron transport chain resulting in the excessive generation of ROS under saline conditions.

### 2.3. Effect of FA on the Oxidative Damage and Cell Membranes Stability Index (CMSI)

**Figure 3:**
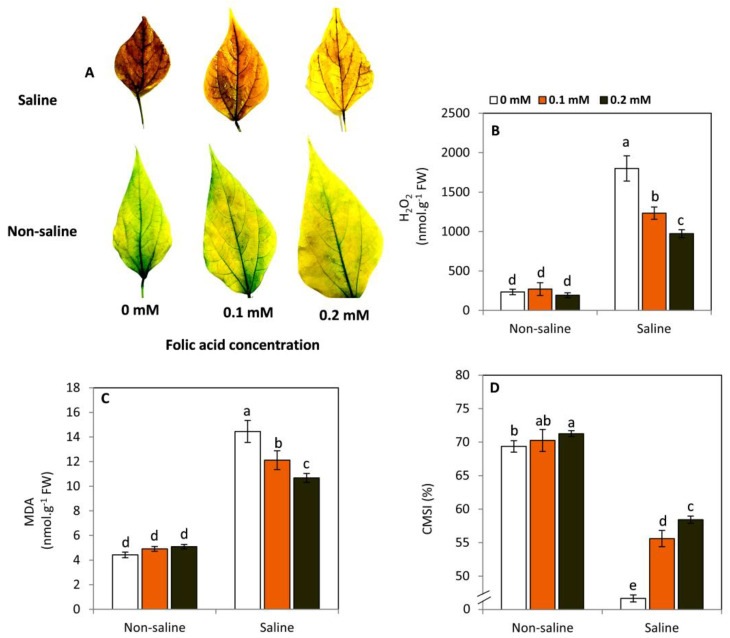
Effect of exogenous folic acid (FA; 0, 0.1, and 0.2 mM) on the histochemical detection of H_2_O_2_ using diaminobenzidine (DAB) staining method (**A**), the concentration of H_2_O_2_ (**B**), lipid peroxidation as indicated by malondialdehyde, MDA (**C**), and cell membrane stability index, CMSI (**D**), of snap bean plants grown under non-stressed and saline-stressed (50 mM, NaCl) conditions. Values are the averages of three replicates ± SD. Different letters indicate significant differences according to Duncan’s multiple range tests (*p* < 0.05). Dark-brown DAB staining color indicates increase the accumulation of H_2_O_2_.

The histochemical detection of H_2_O_2_ by diaminobenzidine (DAB) method (Figure 3A) demonstrated that plants subjected to salt stress (50 mM NaCl) displayed a higher accumulation of H_2_O_2_ in their leaf tissues compared to those of non-saline conditions. This harmful effect was progressively decreased by FA treatments. In this respect, the treatment of 0.2 mM FA was better than the lower concentration. On the other hand, no changes were observed between all treatments under non-saline conditions. Reducing salt stress-induced oxidative damage was also biochemically proven by lowering the concentration of H_2_O_2_ (Figure 3B) and the rate of lipid peroxidation (Figure 3C). These positive responses were reflected in the integrity of cell membranes, as indicated by enhancing CMSI (Figure 3D).

### 2.4. Effect of FA on the Activities of Antioxidant Enzymes

**Figure 4:**
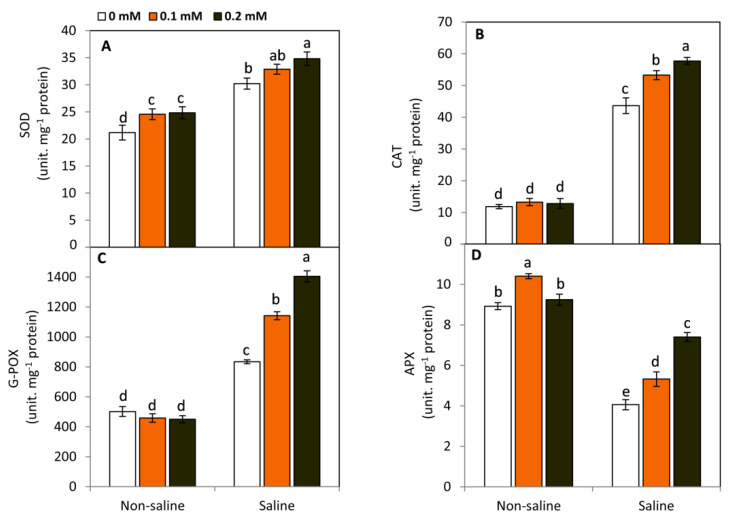
Effect of exogenous folic acid (FA; 0, 0.1, and 0.2 mM) on the activities of antioxidant enzymes including superoxide dismutase, SOD (**A**), catalase, CAT (**B**), guaiacol peroxidase, G-POX (**C**), and ascorbate peroxidase, APX (**D**), of snap bean plants grown under non-stressed and saline-stressed (50 mM, NaCl) conditions. Values are the averages of three replicates ± SD. Different letters indicate significant differences according to Duncan’s multiple range tests (*p* < 0.05).

Plants trigger antioxidant defense systems to avoid the damage of ROS accumulation and lipid peroxidation. Therefore, the activities of antioxidant enzymes including SOD, CAT, G-POX, and APX were determined. In this context, data presented in Figure 4 show that plants exposed to salt stress demonstrated a significant (*p* ≤ 0.05) increase in SOD, CAT, and G-POX activities compared to the unstressed plants. Meanwhile, APX displayed an obvious decrease under saline conditions. Moreover, FA-treated plants exhibited greater improvement in the activities of all studied enzymes compared to untreated plants. In this respect, the treatment of 0.2 mM FA gave the highest significant values in SOD (15.2%), CAT (32.2%), G-POX (68.2%), and APX (82.3%) over the untreated plants under saline conditions. On the other hand, FA-treated plants exhibited a significant increase in SOD (0.1 and 0.2 mM) and APX (0.1 mM) under non-saline conditions.

### 2.5. Effect of FA on RWC and Osmotic Molecules

**Figure 5:**
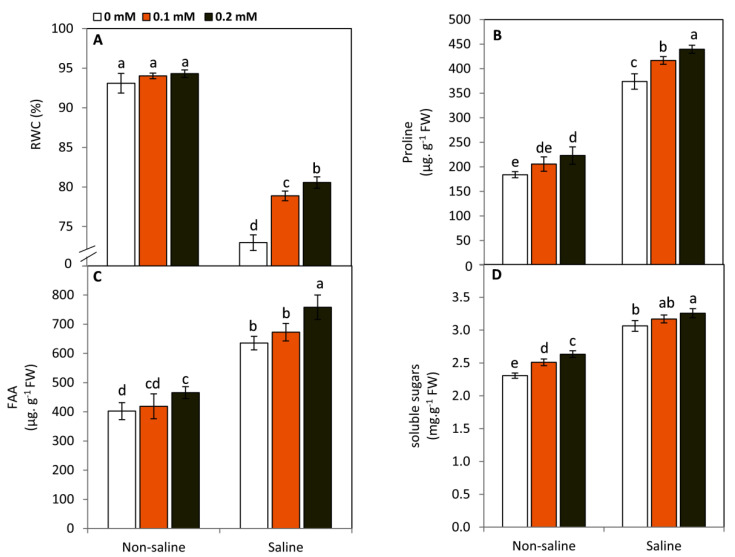
Effect of exogenous folic acid (FA; 0, 0.1, and 0.2 mM) on leaf relative water content; RWC (**A**), proline (**B**), free amino acids, FAA (**C**), and soluble sugars (**D**) of snap bean plants grown under non-stressed and saline-stressed (50 mM, NaCl) conditions. Values are the averages of three replicates ± SD. Different letters indicate significant differences according to Duncan’s multiple range tests (*p* < 0.05).

Plants exposed to saline conditions (50 mM NaCl) showed a significant decrease (*p* ≤ 0.05) in RWC compared to those under non-saline conditions (Figure 5A). Conversely, this negative effect was significantly (*p* ≤ 0.05) reversed by FA treatments. In this respect, the treatment of 0.2 mM FA was more efficient in enhancing RWC than the other treatments. On the other hand, no changes in RWC were observed in FA-treated and non-treated plants under non-saline conditions. In contrast, the osmotic molecules, including proline, free amino acids (FAA), and soluble sugars, demonstrated an enormous accumulation with exposure to salinity stress (Figure 5B–D). The highest significant values in all studied osmolytes were observed in the 0.2 mM FA-treated plants under saline conditions. These increases were amounted by 17.6, 19.3, and 6.3% over the untreated plants in proline, FAA, and soluble sugars, respectively. Moreover, under non-saline conditions, FA-treated (0.2 mM) plants exhibited a significant increase in proline, FAA, and soluble sugars compared to the untreated plants. This response may imply that FA is involved in nitrogen and carbon metabolism regardless of the presence of saline conditions.

### 2.6. Effect of FA on Nutrients

**Figure 6:**
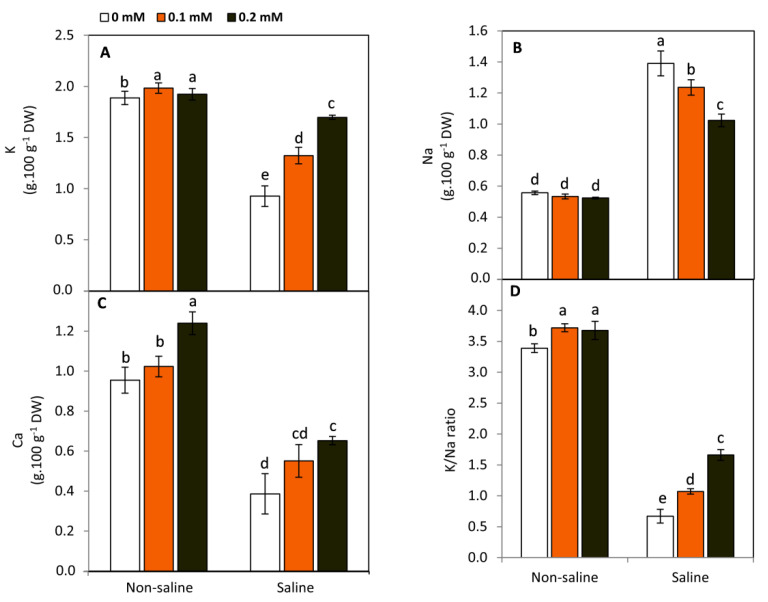
Effect of exogenous folic acid (FA; 0, 0.1, and 0.2 mM) on K (**A**), Na (**B**), Ca (**C**), and K/Na ratio (**D**) in the leaves of snap bean plants grown under non-stressed and saline-stressed (50 mM, NaCl) conditions. Bars represent standard deviation (SD) of the means (n = 3). Values are the averages of three replicates ± SD. Different letters indicate significant differences according to Duncan’s multiple range tests (*p* < 0.05).

Plants exposed to salinity stress demonstrated a significant (*p* ≤ 0.05) decrease in K, Ca, and K/Na ratio compared to the non-saline conditions, while Na revealed an obvious and significant increase in the salt-stressed plants (Figure 6). However, FA significantly hindered the uptake of Na and enhanced the content of K and Ca, leading to an increase K/Ca ratio. These findings may disclose the crucial role of FA in protecting the cytosolic enzymes from the high levels of Na and regulating osmotic potential under saline conditions.

### 2.7. Effect of FA on the Relative Expression of SOS1, NHX1, and Osmotin

**Figure 7:**
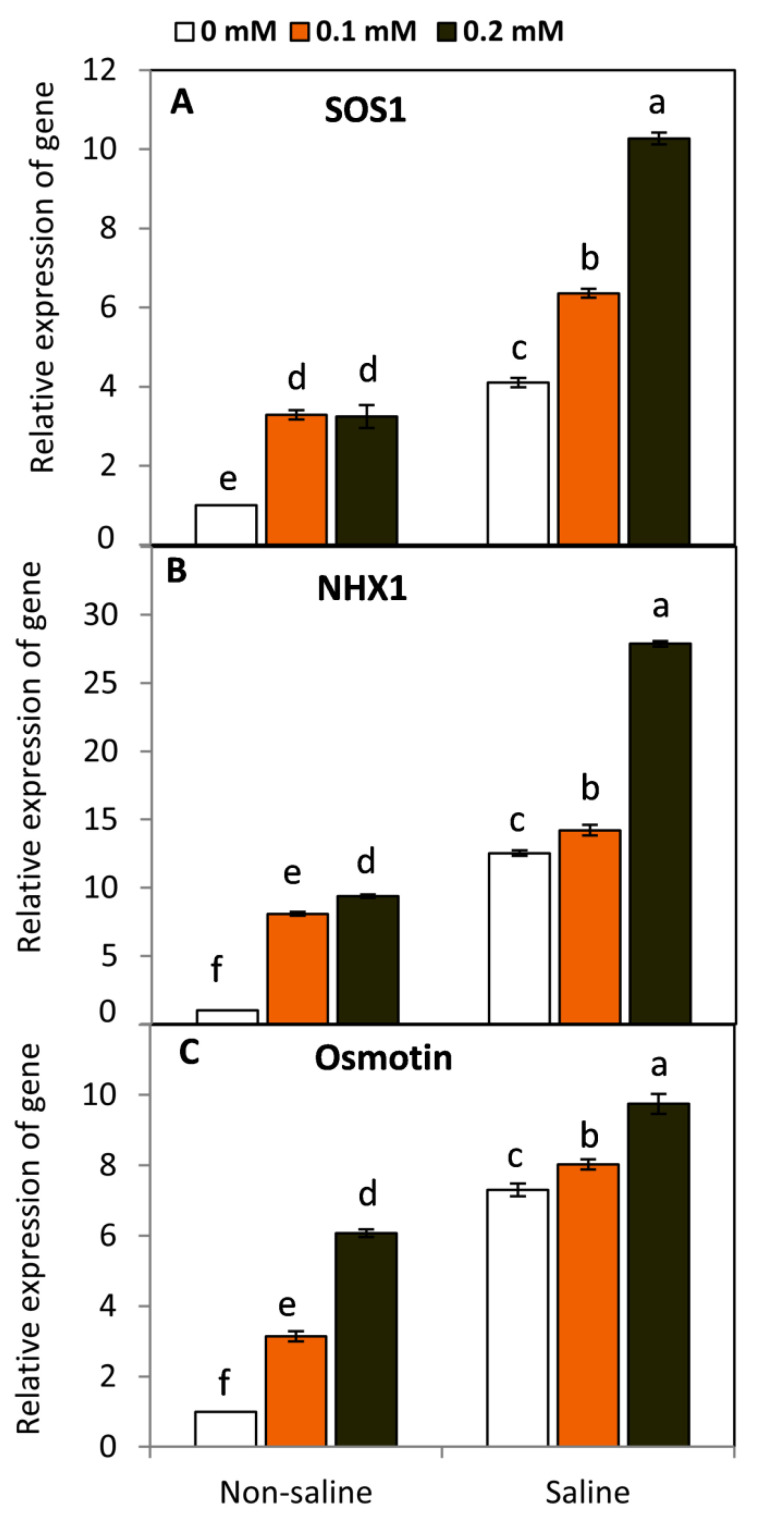
Effect of exogenous folic acid (FA; 0, 0.1, and 0.2 mM) on the relative expression of plasma membrane Na^+^/H^+^ antiporter protein of salt overly sensitive gene (*SOS1*) (**A**), vacuolar-localized Na^+^/H^+^ antiporter protein (*NHX1*) (**B**), and the multifunctional osmotic protective protein (*Osmotin*) (**C**) of snap bean plants grown under non-stressed and saline-stressed (50 mM, NaCl) conditions. Values are the averages of three replicates ± SD. Different letters indicate significant differences according to Duncan’s multiple range tests (*p* < 0.05).

Under saline conditions, the molecular studies using qRT-PCR demonstrated that plasma membrane Na^+^/H^+^ antiporter protein of salt overly sensitive gene (*SOS1*), vacuolar-localized Na^+^/H^+^ antiporter protein (*NHX1*), and the multifunctional osmotic protective protein (*Osmotin*) revealed greater and significant (*p* ≤ 0.05) up-regulation compared to the salt-unstressed plants (Figure 7). Moreover, FA-treated plants exhibited a significant increase in the relative expression of *SOS1, NHX1*, and *Osmotin* compared to the untreated plants either under saline or non-saline conditions. Generally, FA at 0.2 mM under saline conditions gave the highest significant gene expression of *SOS1* (250%), *NHX1* (222%), and *Osmotin* (233%), respectively, compared to the untreated plants.

## 3. Discussion

Plant growth and development of snap bean plants can be seriously affected under saline conditions [36,37,38]. In this study, we observed that exposed plants to salt stress led to an obvious and significant (*p* ≤ 0.05) inhibition in their growth rate as a result of reducing the fresh ad dry weight of the shoot and root systems. Generally, salt stress can restrict plant growth by affecting cell division [39], photosynthesis [11], plant water relations [40], homeostasis of plant hormones [41], and uptake of essential nutrients [42,43]. In contrast, FA significantly (*p* ≤ 0.05) improved plant growth of saline- and non-saline-stressed plants compared to the FA-untreated plants. It is well documented that FA can be involved in the mitigation of the cytotoxic effects of salinity stress [44], biosynthesis of nucleic acids [45], and enhancing various morphological and anatomical aspects under salt stress [28]. In addition, FA regulates many cellular and molecular events that can affect plant growth and development i.e., cell division, genome stability, and gene expression [18,46,47].

Under salinity stress, several changes in the content of Chl a, Chl b, and carotenoids can be observed [7,8,9,11,48]. In this study, salt-stressed plants revealed a significant decrease in Chl a, Chl b, and carotenoids. This effect could be attributed to the presence of oxidative damage responsible for destroying chloroplast membranes [49]. However, FA-treated plants demonstrated an obvious improvement in the content of photosynthetic pigments. Folates are involved in the biosynthesis of chlorophyll and carotenoids due to their participation in the biosynthesis of porphyrins and isoprenoids [17,24]. Furthermore, FA was effective only on Chl b, and not on Chl a, under saline conditions. This response can be considered an important protective and regulatory step to avoid the excessive generation of ROS due to exceeding light absorbance and the over reduction of the photosynthetic electron transport chain [50].

Moreover, it has been found that exogenous FA can improve cell membrane stability index and decrease the oxidative damages caused by osmotic stress induced by drought [24] and high salinity [28]. In this study, FA obviously reduced the accumulation of H_2_O_2_ and lipid peroxidation of salt-stressed plants. Meanwhile, a noticeable improvement in cell membrane stability index was observed in parallel with the increase in FA concentration. Folic acid and its derivatives (folates) can enhance the antioxidant capacity of plants under various stress conditions by stimulating the glutathione-ascorbate cycle [18] and reducing lipid peroxidation [25,26]. Furthermore, FA can be involved in the biosynthesis of the other non-enzymatic antioxidants, such as carotenoids and α-tocopherol (vitamin E), through the synthesis of isoprenoids [17].

The improvement of antioxidant capacity by FA is not restricted to the biosynthesis of non-enzymatic antioxidants, but also by elevating the activities of antioxidant enzymes. In this study, we found that FA significantly (*p* ≤ 0.05) increased the activities of SOD, CAT, G-POX, and APX under saline conditions compared to FA-untreated plants. The integration between the different functions of these enzymes is critically important for plant survival under adverse conditions. Due to the dismutation of superoxide anions by SOD, it is essential to maintain the H_2_O_2_ produced by this reaction under a controlled level in plant tissues [51]. Scavenging of H_2_O_2_ can be enzymatically performed by CAT, APX, and G-POX in diverse pathways. These reactions can catalyze the decomposition of H_2_O_2_ directly (CAT) or indirectly using ascorbate (APX) or phenolic and endolic substances (G-POX) [52,53]. In this study, increasing the activities of CAT, APX, G-POX, and SOD in the FA-treated plants under saline stress may imply its pivotal role in plant tolerance and reducing salt-induced oxidative damage.

Plants exposed to salinity stress exhibited a significant decrease in RWC, while the osmotically active molecules, including FAA, proline, and soluble sugars, revealed considerable accumulation compared to the unstressed plants. Among the negative effects of higher amounts of salts in the soil, the osmotic stress and the inability of the plant to absorb water are considered major constraints to plant growth and productivity [11,42,43]. The accumulation of osmotic molecules can maintain the osmotic potential of the stressed plant cells, leading to absorbing more water and protecting the cytosolic enzymes from ion toxicity [43,53,54]. Folic acid is involved in the biosynthesis of a broad spectrum of amino acids such as glycine, tryptophan, methionine, valine, and proline [17]. Under unfavorable conditions, FA can stimulate the biosynthesis of these organic molecules via the glutamate pathway [17,24]. Additionally, FA can affect the soluble sugars by enhancing photosynthesis and regulating carbon metabolism [18,55].

Under non-saline conditions, FA-treated plants tended to increase the concentration of K and Ca in leaf tissues, while no changes were detected in Na. Thus, these responses led to an increased K/Na ratio in the FA-treated plants compared to the untreated ones. Conversely, under saline conditions, FA-treated plants showed a significant (*p* ≤ 0.05) decrease in Na, while K, Ca, and K/Na ratios were dramatically increased. Salt stress can affect plant water status, leading to modifying the ability of the root system to uptake the different nutrients [11]. Moreover, reducing water uptake can restrict transpiration and nutrient transport via xylem tissue [56]. In this study, FA-treated plants exhibited an obvious increase in the K/Na ratio. Generally, increasing the uptake of K and Ca and decreasing Na under salinity stress are common protective features in salt-tolerant plants. This response implies that FA positively affected the integrity of cell membranes and their nutrient selective permeability.

Under saline conditions, the qRT-PCR analysis demonstrated that the plasma membrane Na^+^/H^+^ antiporter protein encoded by the *SOS1* gene, the tonoplast-localized Na^+^/H^+^ antiporter protein (*NHX1*), and the multifunctional osmotic protective protein (*Osmotin*) revealed greater and significant (*p* ≤ 0.05) up-regulation compared to the salt-unstressed plants. Overexpression of these genes can increase plant tolerance to salt stress by maintaining a higher K^+^/Na^+^ ratio and achieving improved osmotic balance [11,57]. On the other hand, FA-treated plants exhibited a significant increase in the relative expression of *SOS1, NHX1*, and *Osmotin* compared to the untreated plants under saline conditions. This response may refer to its protective role as an efficient antioxidant and vital biomolecule in regulating plant tolerance to salinity stress.

## 4. Materials and Methods

### 4.1. The Treatments and Growth Conditions

Seeds of snap beans (*Phaseolus vulgaris*; cv. Bronco) were surface sterilized before sowing using a solution of 0.5% NaOCl for 3 min, then washed four times with distilled water. Dark grey plastic pots with a diameter 13 cm and a volume 700 cm^3^ were filled with pre-washed sand. Seeds were sown in the pots (one plant/pot) and regularly irrigated with ½ strength Hoagland’s solution every 2 days. All pots were placed under growth chamber conditions (14 h light at 25 ± 2 °C and 10 h dark at 20 ± 2 °C, light intensity 180 µmol m^−^^2^ s^−1^, and 70% relative humidity). Two-week-old seedlings with homogenous size and form were divided into two major groups. One of them was continuously irrigated with ½ strength Hoagland’s solution, while the other group was irrigated with ½ Hoagland’s solution modified by adding 50 mM NaCl to apply the salinity stress. Under each major group, pots were divided into three subgroups to use the folic acid (FA; Bioworld, Dublin, OH, USA) at 0, 0.1, and 0.2 mM treatments. Seedlings were sprayed five times with FA at 15, 20, 25, 30, and 35 days after sowing. The volume of sprayed solution was 10 mL to each plant every single time. Tween-20 as a non-ionic surfactant was used at 0.05% (*v*/*v*) with all foliar treatments. Plants were left to grow for 5 days and then gathered to determine different biochemical and molecular studied traits. The experiment was designed as a complete randomized design (CRD) with three replicates. The total number of pots was 108 (2 salinity × 3 FA x 6 pots × 3 replicates).

### 4.2. Determination of Growth Parameters

After sampling at 40 days, the shoot and root systems of plants (two pots from each replicate) were immediately weighed using a digital balance, whereas dry weight was determined by drying the samples in an air-forced ventilated oven at 70 °C.

### 4.3. Histochemical Detection of H_2_O_2_

The terminal leaflet of the third leaf from the top was ditched to explore histochemically the presence of H_2_O_2_ by the diaminobenzidine (DAB) method [58]. The ditched leaflet from each treatment was completely soaked in a petri dish containing a solution of 100 ppm DAB and 50 mM Tris-HCl buffer, pH 4.0, for 24 h. After that, the leaflet was transferred to absolute alcohol several times to remove the leaf pigments. As much as the dark brown color appeared, the accumulation of H_2_O_2_ in leaf tissues increased.

### 4.4. Determination of H_2_O_2_ and Lipid Peroxidation

The quantification of H_2_O_2_ was done using the method of Velikova et al. [59] with some modifications. Briefly, 0.2 g of leaf tissue was ground in 4 mL tri-chloroacetic acid (TCA) and centrifuged at 10,000 rpm and 4 °C for 15 min. In total, 0.5 mL of the supernatant was added to an equal volume of potassium phosphate buffer (10 mM, pH 7) and 2 mL potassium iodide (1 mM). The absorbance of the developed yellow color was recorded at 390 nm. Malondialdehyde (MDA) was determined using the thiobarbituric acid (TBA) method by reading the absorbance at 535 and 600 nm to correct the non-specific turbidity as described by Heath and Packer [60].

### 4.5. Determination of Cell Membranes’ Stability Index (CMSI)

As described by Abd Elbar et al. [61], cell membrane stability was estimated with some modifications. Eight leaf discs with 1.8 cm diameter were incubated in 10 mL deionized water for 24 h on a shaker. After that, EC_1_ values were measured by EC meters (DOH-SD1, TC-OMEGA, USA/Canada). Then, samples were autoclaved at 120 °C for 20 min to determine the values of EC_2_. The cell membrane stability index was calculated using the following equation:

$$MSI=[1-\left(\frac{EC_{1}}{EC_{2}}\right)]\times 100$$

where EC_1_ is the reading of EC-meter (DOH-SD1, TC-OMEGA, Canada) for 10 leaf discs incubated in 10 mL deionized water for 24 h. Meanwhile, EC_2_ is the reading of the EC-meter for the same leaf discs after membranes’ destruction in the autoclave at 120 °C for 20 min.

### 4.6. Determination of Antioxidant Enzyme Activities

In total, 0.5 g of leaf samples were ground with liquid nitrogen and mixed in a 5 mL extraction buffer (pH 7.0) containing 1 M phosphate buffer and 0.5 mM EDTA. The homogenates were centrifuged at 10,000 rpm for 15 min at 4 °C. The supernatant was used for the determination of enzyme activities. Total soluble protein was determined in the enzyme extract according to Bradford [62]. Catalase (CAT; EC 1.11.1.6) activity was determined by measuring the disappearance of H_2_O_2_ [63]. 0.5 mL of 75 mM H_2_O_2_ was added to 1.5 mL of 0.1 M phosphate buffer (pH 7) and 50 µL of diluted enzyme extract in a 3 mL reaction mixture. The decline of absorbance at 240 nm was measured for 1 min, and enzyme activity was computed by calculating the amount of decomposed H_2_O_2_. Superoxide dismutase (SOD; EC 1.15.1.1) activity was determined by measuring the inhibition of nitro blue 135 tetrazoliums (NBT) at a wavelength of 560 nm [64]. The reaction mixture of the samples included: 50 mM phosphate buffer (pH = 7), NBT 0.075 μM, 0.1 mM Na-EDTA, 75 μM riboflavin, 13 mM methionine, and 50 μL of enzyme extract. Ascorbate peroxidase (APX; EC 1.11.1.11) activity was determined based on the decrease in ascorbate at 290 nm [65].

### 4.7. Quantification of Leaf Relative Water Content (RWC) and Osmotic Molecules

Relative water content (RWC) was estimated by the method of Smart and Bingham [66]. Briefly, 10 fresh leaf discs were precisely weighed (FW) and kept for 1 h in distilled water to gain the turgidity (TW). Then, the leaf discs were oven-dried at 80 °C for 24 h to record the dry weight (DW). The calculation of RWC was done using the following formula:

$$RWC\;\left(\%\right)=\frac{FW-DW}{TW-DW\;}\times 100$$

where FW is the fresh weight of leaf discs, DW is the dry weight, and TW is the turgid weight. Free amino acids (FAA) were determined by ninhydrin reagent as glycine according to the method of Hamilton et al. [67]. Total soluble sugars were estimated by the phenol-sulfuric acid method as described by Chow and Landhäusser [68]. Proline was measured using the ninhydrin reagent, as described by Bates et al. [69].

### 4.8. Estimation of Nutrients (K, Na, Ca, and K/Na Ratio)

The concentration of K, Na, and Ca was determined using the flame photometric method (Jenway, Staffordshire, UK), as described by Havre [70].

### 4.9. Expression of Salt Responsive Genes

According to the manufacturer’s protocol, the total mRNA from different treatments was extracted using 0.5 g of fresh leaves by RNA extraction kit (Sigma-Aldrich). The quality of purified RNA was quantitated using NanoDrop™ 2000/2000c Spectrophotometers, after the reverse transcription of RNA and cDNA formation according to the manufacturer’s protocol (Promega, Walldorf; Germany). Real-time quantitative Reverse Transcription-Polymerase Chain Reaction (qRT-PCR) analysis (Rotor-Gene 6000, Germany) was performed using real-time PCR on 1 L diluted cDNA in triplicate and the primer sequences used in qRT-PCR are provided in Supplementary Table S1. The GAPDH housekeeping gene (reference gene) was utilized to analyze gene expression using SYBR^®^ Green. The relative gene expression was determined using the 2^−ΔΔCt^ method [71].

### 4.10. Statistics

Data of the present study were analyzed by one-way ANOVA using SAS [72] software. Means of three replicates ± SD were calculated. The significant differences between means were estimated using Duncan’s multiple range test at *p* ≤ 0.05.

## 5. Conclusions

**Figure 8:**
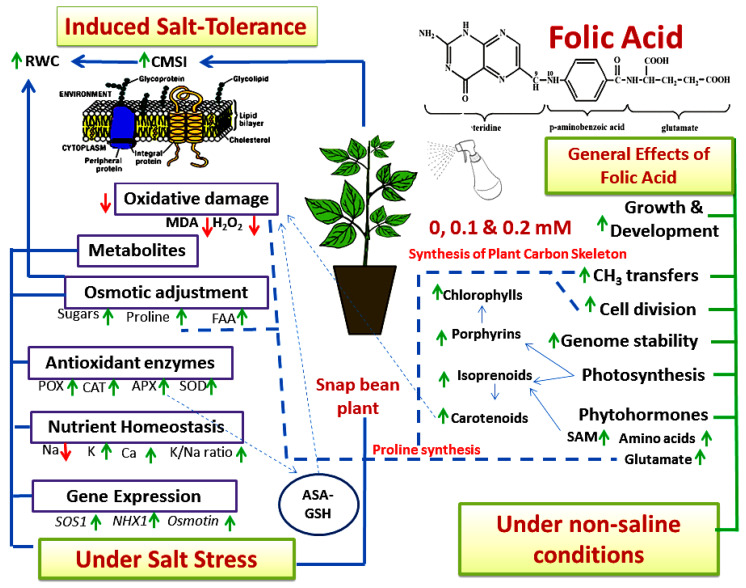
Simplified model for the suggested effect of exogenous folic acid as a foliar spray on snap bean plants grown under salinity stress. RWC, relative water content; CMSI, cell membrane stability index; SAM, S-adenosylmethionine; MDA, malondialdehyde; FAA, free amino acids; POX, peroxidase; CAT, catalase; APX, ascorbate peroxidase; SOD, superoxide dismutase; ASA-GSH, ascorbate glutathione cycle; green upward arrow, increase; red downward arrow, decrease.

Folic acid (FA) is an important water-soluble vitamin (B9) with multiple crucial roles in plants. It serves as a cofactor in the methyl transfer reactions, cell division, DNA synthesis, genome stability, and synthesis of protein and carbohydrates. Moreover, it possesses a high antioxidant capacity, enabling it to serve as ROS scavenger and to reduce the biotic and abiotic stress-induced oxidative damage. Under salt stress conditions, we observed that exogenous FA significantly enhanced CMSI, RWC, FAA, proline, total soluble sugars, G-POX, CAT, APX, SOD activities, K, Ca content, and K^+^/Na^+^ ratios. These effects were associated with enhanced photosynthetic pigments (chlorophylls and carotenoids) and reduced the oxidative damage as indicated by reduced amounts of cytotoxic molecules, such as H_2_O_2_ and MDA. Additionally, salt stress responsive genes including *SOS1*, *NHX1,* and *Osmotin* revealed greater relative expression in the FA-treated plants compared to the untreated ones. In conclusion, the results of this study may imply that exogenous FA has several possible protective effects on salt-stressed snap bean plants. Furthermore, it can be recommended to mitigate the harmful effects of salinity stress. Finally, the mechanisms of stress mitigation by FA in snap bean plants under non-saline and salinity stress conditions can be summarized, as shown in Figure 8. Further studies can be carried out to get a comprehensive picture of the effects of FA on different plant species under various biotic and abiotic stresses.

## Data Availability

Not applicable.

## Supplementary Materials

The following supporting information can be downloaded at: https://www.mdpi.com/article/10.3390/plants11111459/s1, Table S1: Oligonucleaotides primer pairs used for quantitative RT-PCR analysis.
