# Supplementary material for: Folic Acid Confers Tolerance against Salt Stress-Induced Oxidative Damages in Snap Beans through Regulation Growth, Metabolites, Antioxidant Machinery and Gene Expression
Source: Plants (Basel). 2022 May 30;11(11):1459. doi: 10.3390/plants11111459 (PMC9182733; doi:10.3390/plants11111459)
Supplement: Supplementary file 1 [file plants-11-01459-s001.zip › plants-1731093-supplementary.pdf]

Supplementry

Table S1. Oligonucleaotides primer pairs used for quantitative RT-PCR analysis.

Gene Name		Sequence
SOS1	F	5′-ACTTGCAGGAGGAATACAAC-3′
	R	5′- CGAGAAGAGAAGACCACATC-3′
Osmotin-like protein (Osmotin-34)	F	5′-GAACGGAGGGTGTCACAAAATC-3′
	R	5′-CGTAGTGGGTCCACAAGTTCCT-3′
NHX1	F	5′-CGTGATGTCGCATTACACCT-3′
	R	5′- CTGGCAAACTCCCACTTCTC-3′
GAPDH	F	5′-TGACGACATCAAGAAGGTGGTG-3′
	R	5′-:GAAGGTGGAGGAGTGGGTGTC-3′
